# Validation of the Persian version of Skindex-16 among older patients with skin diseases

**DOI:** 10.1186/s12877-021-02635-7

**Published:** 2021-12-18

**Authors:** Tahereh Mahdavi nejad, Fatemeh Mohammadi, Ozkan Gorgulu, Seyedeh Ameneh Motalebi, Zahra Hosseinkhani

**Affiliations:** 1 Student Research Committee, School of Nursing and Midwifery, Qazvin University of Medical Sciences, Qazvin, Iran; 2 Social Determinants of Health Research Center, Research Institute for Prevention of Non-Communicable Diseases, Qazvin University of Medical Sciences, Qazvin, Iran; 3 Department of Biostatistics and Medical Informatics, Faculty of Medicine, Ahi Evran University, Kırşehir, Turkey; 4 Metabolic Diseases Research Center, Research Institute for Prevention of Non-Communicable Diseases, Qazvin University of Medical Sciences, Qazvin, Iran

**Keywords:** Aging, Health-related quality of life, Skindex-16, Validation

## Abstract

**Background:**

Skin conditions often considerably impact the older patients’ psycho-social health and quality of life (QoL). The present study was aimed to examine the validity and reliability of the Persian version of Skindex-16 among older people with skin diseases.

**Methods:**

In this validation study, 260 older patients suffering from a range of skin conditions were recruited from a dermatology clinic in Rasht, Iran. Data were collected using a checklist for demographic and clinical characteristics and the Skindex-16 questionnaire. In this study, validity (face, content, and construct) and reliability (Cronbach’s alpha) of the Skindex-16 were assessed and reported.

**Results:**

The mean age of participants was 64.51 ± 5.04 years. The results of confirmatory factor analysis showed that the model had acceptable fitness into the expected three-factor structure [χ 2 /df = 249.363, *P* < 0.001; GFI = 0.961; TLI =0.952; RMSEA = 0.078 (90% CI = 0.06, 0.09) and SRMR = 0.06]. The reliability analysis results confirmed that the values of Cronbach’s alpha coefficient for Skindex-16 were in the acceptable range (0.923).

**Conclusions:**

Our evaluation of the Skindex-16 indicates that it is reliable and a valid instrument that can be used for measuring QoL for Iranian dermatologic patients.

## Introduction

Aging is associated with a decline in physiological functions, functional capacity, and the onset of many diseases [1]. Like other biological systems, the skin, which provides a large body interface with the environment, grows, matures, and ages throughout life [2]. With advancing age, the prevalence of some skin disorders increases [3]. Skin aging is accompanied by a decrease in the functional capacity that increases the vulnerability to cutaneous problems and the subsequent development of dermatoses such as xerosis, pruritus, and eczema, and skin cancers [4]. The most common skin disorders in older adults consisted of dermatitis, fungal infections, pruritus, benign and malignant tumors [2]. In a review study of 4099 elderly patients, five common skin diseases were eczema dermatitis (20.4%), fungal infections (15.8%), pruritus (11.5%), and bacterial (7.3%) and viral (6.7%) infections, respectively [5]. In Iran, Morowatisharifabad et al. [6] reported that the most frequent skin problem among older people was dry skin (30.25%) followed by skin pruritus (9.5%), and flaking of the skin (8.25%). Malekzad et al. (2007) [7] also found that 267 out of 456 institutionalized older adults in Iran (58.6%) suffered from at least one type of skin disease. Dry skin, seborrheic keratosis, and pressure ulcers were the most common skin disorders among them [8].

Studies of quality of life (QoL) of patients with skin diseases becoming more important [9]. Chronic inflammatory skin diseases affect the physical, social, and psychological aspects of a person’s life [10, 11]. Severe skin diseases and the presence of symptoms such as itching and pain can result in significant psychosocial consequences [12]. Comorbid psychiatric conditions, including depression, anxiety, and social panic, are often associated with the itch and scratch cycle [13]. Embarrassment due to deformity of the skin can deteriorate the self-esteem and self-confidence of patients and may lead to social isolation [14]. The impact of skin conditions on social relationships might be far more problematic than its related physical limitations [15]. So, it is suggested to consider improving all aspects of the health of these patients. Health-related QoL (HrQoL) is an indicator that can reflect these aspects [16]. Furthermore, the HrQoL assessment provides homogeneous information for the impacts of different skin diseases, as well as the outcomes of different treatments [17].

Culture is an important factor that impacts the perception of QoL among patients with skin diseases. In Iran, cultural aspects such as Islamic clothing can actually ease or exacerbate the symptoms of skin diseases. Wearing particular types of clothes can aggravate skin irritation and trigger flare-ups associated with psoriasis. On the contrary, clothing prevents direct exposure to sunlight and masks symptoms such as red and dry skin, and can improve social aspects [18]. Furthermore, cultural beliefs may increase the distress of patients with skin diseases. For example, Iranian married women with developing vitiligo after marriage may have marital problems that might lead to divorce [19].

Given the impact of skin diseases have on the QoL of older people [20], an accurate, sensitive instrument is required to measure the HrQoL of this group of patients. To address this need, several instruments have been developed to measure the HrQoL of patients with skin problems include Skindex-29 [21], Douleur Neuropathique 4 (DN4), 12-item General Health Questionnaire (GHQ-12) [22], Skindex-16 [23], and Dermatology Life Quality Index (DLQI). DN4 is one of the questionnaires that can be useful in the assessment of neuropathic pain. This questionnaire with 10 items is very specific and only assesses the quality of pain [24]. GHQ-12 is a screening tool to detect mental disorders in the general population and it has not been used specifically for a group of patients [22]. DLQI is the only disease-specific quality of life instrument that was validated among Iranian patients with vitiligo and psoriasis [25, 26].

The Skindex-16 is a commonly used tool that was designed by Chren et al. [23] to measure the effect of skin diseases on the QoL of patients. An original 62-item questionnaire [21] was modified to 29-items [27], and finally to 16 items [23]. It is a self-administered questionnaire that was originally created in English and measures the impacts of skin disease on a patient’s QoL. The Skindex-16 has advantages over the Skindex-29, in that it is a brief, single-page version with fewer items [28]. Adaptations of Skindex-16 have already been made into several languages, including English [23], Italian [29], Arabic [30], Chinese [31], Japanese [32], Moroccan Arabic [33], and Brazilian [34]. In Iran, Soghi et al. [35] translated the scale into Persian based on World Health Organization protocol and assessed content validity, inter-item consistency, and test-retest reliability among a group of Iranian patients with Neurofibromatosis Type 1. However, due to the significant role of culture and context in perceiving pain, physical and psychological symptoms [36], and QoL [37], it is necessary to validate Skindex-16 in Iran. Therefore, this study aimed to determine the psychometric properties (face, content, construct validity, and internal reliability) of the Persian version of the Skindex-16 that was previously translated by Soghi et al. [35] in a sample of older patients with skin diseases.

## Methods

The study sample consisted of 260 older patients with skin disease recruited via convenience sampling method from a dermatology clinic of Razi hospital, Rasht, Iran. Inclusion criteria were age 60 years old or over and a diagnosis of dermatological disease. Participants who were unable to communicate verbally were excluded from the study.

The minimum sample size required for conducting factor analysis is equal to five to ten subjects per item [38]. Furthermore, the general rule suggested by Boomsma and Hoogland [39] for factor analysis is using samples greater than 200.

Given the expected response rate from 70 to 80% due to conditions such as culture, education, and response quality, We recruited a total of 325 older patients in the study. However, as expected, 20% of responses did not meet the quality assessment, thus, a sample size of 260 was utilized to report the findings from the study.

The data collection was lasted 2 months, from 20th of April to 20th of Jun 2020. A trained reviewer (first author) gathered the data by face-to-face interview.

### Instruments

Data were collected using a demographic and clinical characteristics checklist and the Skindex-16 questionnaire.

A demographic and clinical characteristics checklist was used to collect information about the older patients’ age, gender, marital status, education level, job, living arrangement, history of psychological or physical problems, type of skin disease, duration of skin disease, and duration under treatment.

The Skindex-16 was developed by Chren et al. [23] to measure the impact of skin diseases on HrQoL, has been translated and validated in several languages and it is the most commonly used instrument to measure HrQoL in patients with skin diseases. The Skindex-16 consists of 16 items which are scored on a seven-point Likert scale, from 0 (never bothered) to 6 (always bothered). The range of possible scores was from 0 to 96, where lower scores indicate higher levels of QoL. In this study, we used the Persian version of the Skindex-16, which was translated based on the World Health Organization protocol in a previous study [35].

Access to the questionnaire, or permission to use the Skindex-16, can be found on https://eprovide.mapi-trust.org/instruments/skindex.

### Face validity assessment

For the face validity, ten older patients were asked about the appropriateness of the items of the instrument to measure the targeted construct. They were also asked about the level of difficulty, the degree of inconsistency, ambiguity of expressions [40]. The items of the scale were corrected according to their comments.

### Content validity assessment

The content validity of the Persian version of the Skindex-16 was evaluated by qualitative and quantitative methods. For the qualitative content validity, the expert panel was defined as individuals who were experts in dermatology, gerontology, psychology, and educational nursing. They were asked to give feedback on the items of the Skindex-16 scale in terms of relevancy, understandability/clarity wording, item allocation, grammar, and scaling [41]. A final version of the scale was created after applying their comments.

The Content Validity Ratio (CVR) and the Content Validity Index (CVI) were calculated for the quantitative content validity analysis. In this way, 15 experts were asked to rate the essentiality of the Skindex-16 items on a 3-point Likert-type scale consisting of “not essential”, “useful but not essential”, and “essential” [40, 42]. Then the questionnaire’s CVR was assessed; according to the Lawsche table, if the item score was over 0.49, the item was considered as an appropriate and necessary one [43]. For the Item-CVI (I-CVI), the value of 0.79 or greater shows the item to be appropriate [44] The CVR and I-CVI for the items of Skindex-16 are presented in Table 1.

**Table 1:** The I-CVI and CVR for the Skindex-16 Scale items

Items	I-CVI	CVR
	Relevancy (1–4)	Essential (1–3)
1. Your skin condition itching	1.00	1.00
2. Your skin condition burning or stinging	1.00	0.86
3. Your skin condition hurting	0.93	0.60
4. Your skin condition being irritated	0.80	0.60
5. The persistence/reoccurrence of your skin condition	0.93	0.73
6. Worry about your skin condition (For example: that it will spread, get worse, scar, be unpredictable, etc)	0.93	0.60
7. The appearance of your skin condition	0.86	0.60
8. Frustration about your skin condition	0.80	0.60
9. Embarrassment about your skin condition	0.80	0.60
10. Being annoyed about your skin condition	0.80	0.73
11. Feeling depressed about your skin condition	0.80	0.60
12. The effects of your skin condition on your interactions with others (For example: interactions with family, friends, close relationships, etc)	1.00	0.73
13. The effects of your skin condition on your desire to be with people	1.00	0.60
14. Your skin condition is making it hard to show affection	0.93	0.60
15. The effects of your skin condition on your daily activities	1.00	0.73
16. Your skin condition making it hard to work or what you do	1.00	0.60

*I-CVI* Items Content Validity Index, *CVR* Content Validity Ratio

### Construct validity assessment

#### Factor structure validity

The results of the previous studies [23, 29–34] have shown a three-factor structure – symptoms (items 1 to 4), emotions (items 5 to 11), and functioning (items 12 to 16) – for Skindex-16. So, in this study, a confirmatory factor analysis (CFA) was carried out to confirm the three-factor structure of Skindex-16 for a sample of older patients with skin diseases. Various indexes to evaluate fit were used: Chi-Square/degrees of freedom (χ^2^/df); an adjusted model showing a value between 2 and 3; goodness of fit index (GFI), values near to 0.90 are recommended; Tucker-Lewis index (TLI), values higher than 0.90 and 0.95 are considered as an indicator of satisfactory and good fit, respectively; standardized root mean square residual (SRMR), values less than 0.05 are considered satisfactory, however values close to 0.08 may also be acceptable; and root mean square error of approximation (RMSEA), values < 0.05 as excellent and < 0.08 as acceptable [45–47].

### Reliability assessment

For reliability analysis, Cronbach’s alpha was determined to assess internal reliability. A coefficient of Cronbach’s alpha greater than 0.70 indicates that the scale is highly reliable [48].

### Ethical consideration

The study was approved by the Ethics Committee of Qazvin University of Medical Sciences, Qazvin, Iran (IR.QUMS.REC.1398.269). Older patients were informed about the objectives and procedures of the study. Furthermore, they were instructed that participation was voluntary and that it would not affect their medical care. The confidentiality of the patients’ information was guaranteed. Informed consent was signed by all the older patients before completing the questionnaires.

### Statistics

The Statistical Package for Social Sciences version 21.0 software for Windows (IBM SPSS Statistics for 265 Windows, Version 21.0. Armonk, NY: IBM Corp., USA) and Mplus (Version 7.3, October 2014) software with maximum likelihood estimation were used for statistical calculations. Descriptive statistics of the variables are presented as mean ± standard deviation and n (%). Descriptive analysis to present population norms and reliability analysis, using Cronbach alpha coefficients, was conducted using the entire sample. The normal distributions of the data were confirmed by skewness and kurtosis. CFA was used for evaluating the construct validity of the Skindex-16.

## Results

A total of 260 patients were enrolled. The mean age (SD) of participants was 64.51 (5.04) years. More than half of the older patients were female (*n* = 148, 56.9%) and illiterate (*n* = 64, 24.6%) or in the elementary educational level (*n* = 72, 27.7%). Demographic characteristics of the sample were summarized in Table 2.

**Table 2:** Demographic characteristics of the sample

Age (years), mean (SD)	64.51 (5.04)
Gender, n (%)	
Female	148 (56.9)
Male	112 (43.1)
Marital Status, n (%)	
Single	43 (16.5)
Married	217(83.5)
Educational level, n (%)	
Illiterate	64 (24.6)
Elementary	72 (27.7)
Secondary	29 (11.2)
Diploma and higher	95 (36.5)
Job, n (%)	
Housewife	109 (41.9)
Retired	76 (29.2)
Employed	62 (23.8)
Unemployed	13 (5.0)
Living arrangement, n (%)	
With spouse	61 (23.5)
With children	21 (8.1)
With spouse and children	153 (58.8)
Other	2 (0.8)
Alone	23 (8.8)
Psychological Problems, n (%)	
No	173 (66.5)
Yes	87 (33.5)
Physical Problem, n (%)	
No	48 (18.5)
Yes	212 (81.5)
Type_of_SkinDisease, n (%)	
Pemphigus	97 (37.3)
Psoriasis	108 (41.5)
Eczema	47 (18.1)
Fungal infection	6 (2.3)
Itching	1 (0.4)
Lupus	1 (0.4)
History Skin Family Disease, n (%)	
No	210 (80.8)
Yes	50 (19.2)
Duration Skin Disease, (years), mean (SD)	11.21 (9.68)
Duration under treatment, (years), mean (SD)	11.04 (9.88)

The Cronbach’s alpha coefficients of Skindex-16 and its scales were high (0.859-0.945), which signified very good internal consistency reliability of instruments (Table 3). According to the results, all of the item-total correlation values are greater than 0.30 that are acceptable [49].

**Table 3:** Descriptive information and Cronbach’s alpha of Skindex-16

Questionnaire	Subdomains	Mean (SD)	Percentile			Corrected item-total correlation	Cronbach’s alpha
			25	50	75		
Skindex-16	Symptoms	14.30 (4.59)	10	14	18	0.578-0.767	0.859
	Emotions	22.87 (8.33)	16	21	29	0.569-0.788	0.904
	Functioning	11.35 (5.33)	7	10	15	0.816-0.882	0.945
Skindex-16		48.51 (14.91)	39	47	57	0.532-0.733	0.923

**Fig. 1:**
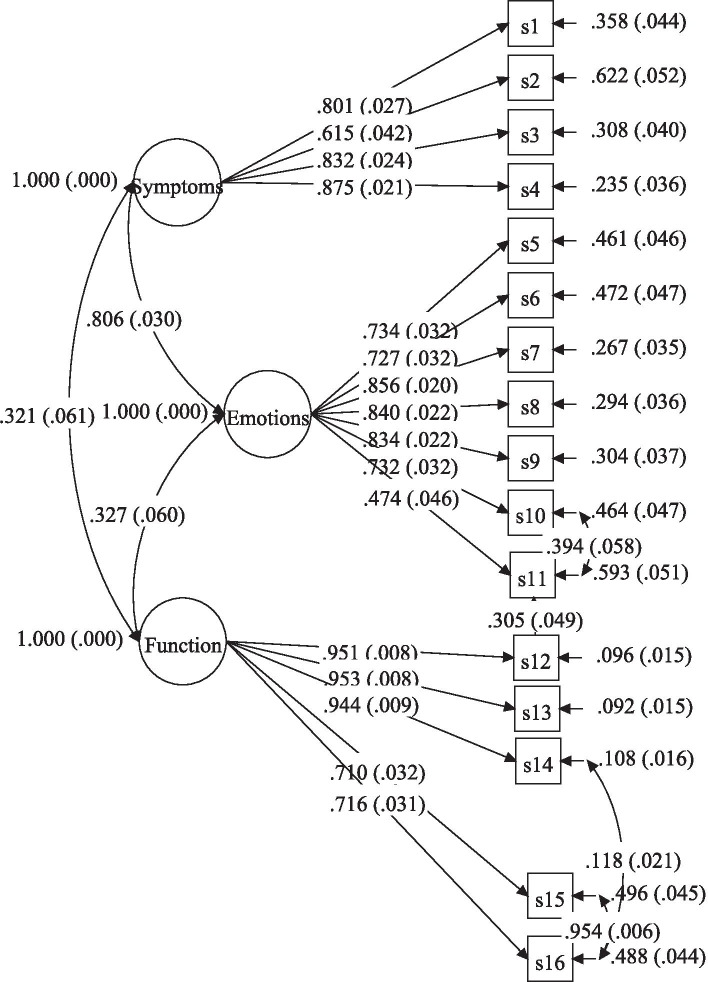
The three-factor model for Skindex-16 obtained from confirmatory factor analysis. Note: f1 = Symptoms, f2 = Emotions, f3 = Functioning

CFA was used to test the construct validity of the Skindex-16. The results of the statistical analysis showed that the model was confirmed in the studied samples [χ 2 /df = 249.363, *P* < 0.001; GFI = 0.961; TLI =0.952; RMSEA = 0.078 (90% CI = 0.06, 0.09) and SRMR = 0.06]. The results indicated to be a goodness of fit indices. The values of two indexes, GFI/TLI and RMSEA were at an acceptable level [50]. The factor loading of all the questions was above 0.6 that is approved [51]. When the fit indexes obtained as a result of CFA were examined, it was determined that the model formed was structurally valid. The structural shape of the model is given in Fig. 1.

## Discussion

*Skin conditions* can significantly influence body image and *psychological* health and consequently, reduce QoL [52]. This study was conducted to determine the psychometric properties of the Persian version of the Skindex-16 among older participants with dermatological conditions referred to Razi Clinic in Rasht, Iran. In order to evaluate the underlying structure of this questionnaire, the CFA method was used. The results of CFA indicated that the three-factor structure of Skindex-16 was acceptable. Results of the current study are in sync with other international studies confirming the three-dimensionality of the scale [23, 29–34]. For example, He et al. (2014) reported the CFA with acceptable fitness into the expected three-factor structure of the Skindex-16 among 225 Chinese patients with skin disease [32]. Furthermore, in a study by Cárcano et al. (2018), an acceptable model fit was obtained for a three-factor structure among 110 Brazilian patients with skin disease [35].

In the present study, one of the factors was the symptoms of skin diseases. Physical impacts of the skin conditions depend on the extent of the rash, how active the rash is, its area, and the related symptoms [53]. Patients with skin disease may experience severe symptoms, such as itching, pain, and discomfort that can have a profound impact on people’s mental health. This most noticeable impact could be due to the visibility of most symptoms, and the influence on daily and work activities [9]. Furthermore, severe skin-related symptoms on the face and body can worsen the self-esteem and self-confidence of dermatologic patients, which significantly affect their social activities involvement [54].

The results CFA showed that the emotional dimension was another acceptable factor of Skindex-16. The burden of skin diseases causes a wide range of unfavorable impacts on the physical, psychological, and social aspects of a patient’s life [52], resulting in impairment in HrQoL. The psychological factor as a general term includes psychological and emotional reactions that occur in a person’s life [55, 56]. Regarding socio-cultural perspective, people judge others based on their physical appearance [57]. Similarly, Rosen and Underwood (2010) found that those who have higher facial attraction are more favourable in comparison with those who have less facial attraction [58]. In the Iranian society, physical attractiveness also plays a crucial role in the social relationships and it is considered as selection criteria for desirability [59]. So, the majority of the Iranian patients with skin diseases suffer from emotional problems due to their chronic nature and its impact on the physical appearance and individuals’ perception of their appearance [35]. Furthermore, there are complex, two-sided associations between psychological factors and dermatological conditions [53]. Psychological stress is related to many common skin diseases and conditions, it seems to be the reason for their onset or aggravation. Psychological impacts related to skin diseases such as fear, mental distress, embarrassment, and depression can affect negatively patients’ QoL [54, 60, 61].

Based on the results CFA, the social performance (functioning) was an acceptable factor of the Skindex-16 scale. Skin-related symptoms not only result in physical discomfort and inconvenience but also influence the personal and social lives of patients [54]. Because the skin is important in human aesthetics and appearance. It is also an important factor in nonverbal communication and interpersonal relations [15]. Many patients limit their physical and social activities, including sports and work, because of reluctance to allow others to see their skin disease [11]. Moreover, severe skin-related symptoms on the face and body can worsen the self-esteem and self-confidence of patients, which significantly affect their involvement in social activities and increase distress [9].

With respect to reliability, the Cronbach’s alpha coefficients for the Skindex-16 and its three dimensions were from 0.859-0.945, which indicated satisfactory internal consistency of the Persian version of the scale. In a study by Chren, Lasek [23] on the original Skindex-16 version, Cronbach’s alpha for the symptoms, emotions, and function were 0.86, 0.93, and 0.92, respectively. In the study of Higaki, Kawamoto [32] the internal reliability of the scale at a high level with Cronbach’s alpha was equal to 0.92. In the study of AlGhamdi and AlShammari [30] which examined the Arabic version of Skindex-16 among the skin patients, the Cronbach’s alpha coefficient for the total instrument was 0.93.

### Limitations

There were some limitations in this study. Since the study was performed among the older patients referred to one dermatological clinic, it may be difficult to generalize the results and state that Skindex-16 is valid and reliable for all Iranian patients with skin diseases. The researchers’ attempt to measure the test-retest reliability of the scale failed, as it was evaluated in an inappropriate time interval (2 weeks). Because the patients were recruited from a dermatological clinic and they underwent the treatments that affected the state of their problems and their QoL was likely to change. Another limitation refers to the response bias, including social desirability bias that seems to be relevant to the geriatric population [62].

## Conclusions

In general, the findings of the present study indicated that the Persian version of the Skindex-16 has a three-factor structure and acceptable validity and reliability, thus justifying its use as a way of evaluating QoL in Iranian older patients with skin conditions. Its shortness and ease of implementation provide researchers and clinicians with a method of readily and extensively using it for QoL assessment in this group of patients.

### Suggestions for future studies

It is suggested that future studies would include patients from multi-center clinics, carry out the same research among patients from different socio-cultural areas and environments, and evaluate the test-retest reliability at an appropriate time interval.

## Abbreviations

QoL: Quality of Life
HrQoL: Health-Related Quality of Life
DN4: Douleur Neuropathique 4
GHQ-12: 12-item General Health Questionnaire
DLQI: Dermatology Life Quality Index
CFA: Confirmatory factor analysis
